# Integrated multi-omics analysis describes immune profiles in ischemic heart failure and identifies PTN as a novel biomarker

**DOI:** 10.3389/fmolb.2024.1524827

**Published:** 2024-12-11

**Authors:** Ting Xiong, Quhuan Li, Yifan Wang, Ying Kong, Hailin Li, Jie Liu, Yueheng Wu, Fengxia Zhang

**Affiliations:** ^1^ School of Bioscience and Bioengineering, South China University of Technology, Guangzhou, China; ^2^ Department of Cardiovascular Surgery, Guangdong Cardiovascular Institute, Guangdong Provincial People’s Hospital (Guangdong Academy of Medical Sciences), Southern Medical University, Guangzhou, Guangdong, China; ^3^ Department of Nephrology, First Affiliated Hospital of Gannan Medical University, Ganzhou, Jiangxi, China

**Keywords:** ischemic heart failure, immune response, multi-omics, biomarker, PTN

## Abstract

**Introduction:**

Heart failure is a leading global cause of mortality, with ischemic heart failure (IHF) being a major contributor. IHF is primarily driven by coronary artery disease, and its underlying mechanisms are not fully understood, particularly the role of immune responses and inflammation in cardiac muscle remodeling. This study aims to elucidate the immune landscape of heart failure using multi-omics data to identify biomarkers for preventing cardiac fibrosis and disease progression.

**Methods:**

We utilized multi-omics data to elucidate the intricate immune landscape of heart failure at various regulatory levels. Given the substantial size of our transcriptomic dataset, we used diverse machine learning techniques to identify key mRNAs. For smaller datasets such as our proteomic dataset, we applied multilevel data cleansing and enhancement using principles from network biology. This comprehensive analysis led to the development of a scalable, integrated -omics analysis pipeline.

**Results:**

Pleiotrophin (PTN) had shown significant upregulation in multiple datasets and the activation of various molecules associated with dysplastic cardiac remodeling. By synthesizing these data with experimental validations, PTN was identified as a potential biomarker.

**Discussion:**

The present study not only provides a comprehensive perspective on immune dynamics in IHF but also offers valuable insights for the identification of biomarkers, discovery of therapeutic targets, and development of drugs.

## 1 Introduction

Heart failure (HF) is a clinical syndrome typically characterized by impaired cardiac pumping and/or filling capacity (Bozkurt et al., 2021). It affects an estimated global population of 60 million annually, resulting in high mortality rates, severe symptoms, and substantial healthcare costs (Savarese et al., 2023). Ischemic heart failure (IHF) has consistently emerged as the primary cause of heart failure owing to the persistent ischemic state of the myocardium, which is often associated with coronary artery disease (Gharbin et al., 2023).

Regulation of the immune response has emerged as a prominent factor in the pathology of IHF, as prolonged ischemia induces hypoxia and persistent inflammatory reactions, subsequently leading to necrosis of the cardiac tissue, triggering ventricular remodeling, which may ultimately result in irreversible damage (Feng et al., 2023; Nian et al., 2023). In the early phase of cardiac remodeling, fibroblasts undergo transformation into myofibroblasts (MFBs) while simultaneously depositing the extracellular matrix (ECM). Dysplastic remodeling of the non-infarcted area consequently induces detrimental alterations in the cardiac tissue and fibrosis may occur independently prior to inflammatory blockade, making it a crucial target for heart failure prevention (Polyakova et al., 2011; Rao et al., 2021). Despite the substantial immune characteristics observed in IHF (Nian et al., 2023; Polyakova et al., 2011; Rao et al., 2021) and the ongoing development of multiple drugs focused on immune inhibition, a sufficient target for preventing myocardial fibrosis has yet to be discovered.

Numerous studies have used sequencing technologies, including transcription, translation, and epigenetics, to investigate IHF. The mechanisms underlying IHF exhibit considerable heterogeneity at various regulatory levels. Research has systematically explored IHF from multiple perspectives ranging from the epigenome to the metabolome (Basak et al., 2015; Kanapeckaitė and Burokienė, 2021; Rong et al., 2022). Some studies have conducted multi-omics analyses to elucidate the intricate landscape of IHF. For example, integrated proteome and metabolome data from ischemic and dilated cardiomyopathy samples have been used to construct a protein–metabolite network (Li et al., 2020). Additionally, a combination of single-nucleus sequencing (snRNA-seq) and bulk RNA-seq has provided a comprehensive cell atlas of IHF on a global scale (Chaffin et al., 2022). These findings offer insights into the diverse biological contexts underlying IHF.

In this study, we conducted a systematic investigation using multi-omics data to discover novel immune markers in IHF that could potentially be targeted for therapy. At the transcriptome level, we identified key immune markers and developed a prediction model through differential expression analysis, enrichment analysis, and machine learning. For proteomic analysis, we conducted protein-protein interaction (PPI) analysis and identified a panel of protein markers by integrating various algorithms and disease-specific profiles. Through the integration of co-expression analysis and experimental validation, we have identified the potential of candidate molecules as a novel biomarker. Our objective was to gain a comprehensive understanding of the immune landscape in IHF, thereby facilitating the development of targeted therapeutic strategies aimed at enhancing diagnostic precision and offering innovative treatment alternatives for patients with IHF.

## 2 Materials and methods

### 2.1 Data acquisition and sample sources

The IHF-associated transcriptome profiles, encompassing the microarray data (GSE5406, GSE57338, GSE1145 and GSE79962), bulk RNA sequencing data (GSE48166, GSE116250, GSE46224 and GSE120825), and single-cell RNA sequencing (scRNA-seq) (GSE121893), with their corresponding probe annotation platforms, were acquired from the Gene Expression Omnibus database (GEO) (Barrett et al., 2013) maintained by the National Center for Biotechnology Information (NCBI, https://www.ncbi.nlm.nih.gov/geo/). Details of the datasets are presented in Supplementary Table S1. The proteome profile associated with IHF, comprising 15 non-failing and six IHF samples from the left ventricular region, was extracted from a study conducted by Barallobere et al. (Barallobre-Barreiro et al., 2021). Additionally, a list of genes related to the immune response was obtained from the ImmPort database (https://www.immport.org/) (Zhou et al., 2019).

**FIGURE 1:**
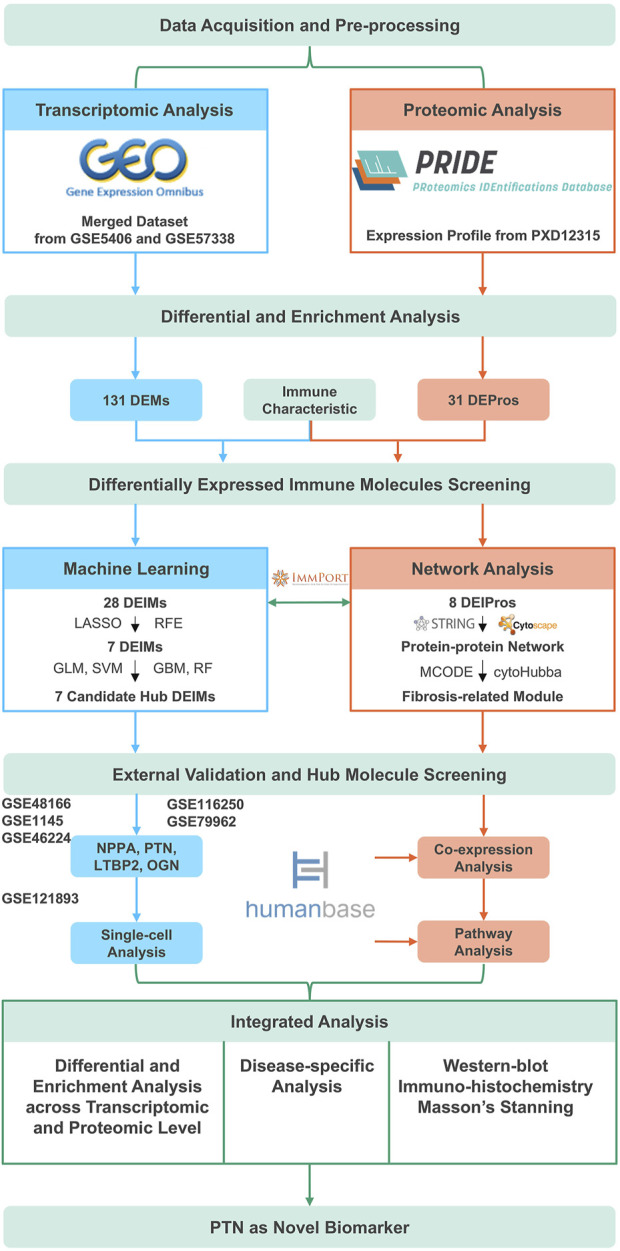
The overall flowchart. DEMs, Differentially expressed mRNAs; DEPros, Differentially expressed proteins; DEIMs, Differentially expressed immune mRNAs; DEIPros, Differentially expressed immune proteins; LASSO, Least absolute shrinkage and selective operator; RFE, Recursive feature elimination; GLM, Generalized linear model; SVM, Supporting vector machine; GBM, Gradient boost machine; RF, Random forest; NPPA, Natriuretic peptide type A; PTN, Pleiotrophin; LTBP2, Latent transforming growth factor beta binding protein 2; OGN, Osteoglycin.

The study was approved by the Ethical Committee of Guangdong General Hospital in Beijing, China. Written informed consent was obtained from all patients, and the experiments were conducted in accordance with the approved study protocol. We analyzed a total of 10 heart samples, including five samples from patients with IHF and five from individuals with healthy hearts. The heart samples used in this study were exclusively sourced from the heart bank of Guangdong General Hospital. Specifically, ischemic cardiomyopathy heart samples were procured from failing hearts acquired during heart transplantation, while samples from healthy hearts were sourced from donor organs that were not used for transplantation due to non-cardiac factors. A flowchart of the study is presented in Figure 1.

### 2.2 The process of data preprocessing

Reference human genome and annotation files (GRCh38) were obtained from the Ensembl database (https://www.ensembl.org) (Cunningham et al., 2022). The probe ID of the microarray dataset was converted to gene symbols based on GPL annotation. Raw RNA-seq data were extracted from the European Nucleotide Archive (ENA, http://www.ebi.ac.uk/ena) and subsequently converted into counts. FastQC was used to assess the quality of the raw reads, whereas Trimmomatic software was used to filter out adaptors and low-quality bases. Clean reads were aligned to the reference genome using HISAT2 (Kim et al., 2015). SAMtools was used to convert the sequence-mapping format into a binary mapping format. Additionally, HTseq 2.0 software was used to obtain counts. The expression profile and phenotype file for the dataset GSE121893 were loaded using the Seurat package (Hao et al., 2021), with SCTransform used for standardization. The “vars.to.regress” = “nCount_RNA” parameter was set to correct expression values. To increase the sample size for subsequent analysis, the GSE5406 and GSE57338 datasets were merged and batch effects were removed using the removeBatchEffect function in the limma package (Ritchie et al., 2015). Principal component analysis (PCA) was performed using the PCA function in the factoextra package.

### 2.3 Identification and functional enrichment analysis of differentially expressed mRNA or proteins

The DESeq2 package was used to analyze the differential expression levels of mRNAs in IHF (Love et al., 2014), using the threshold criteria of |log2 fold change, FC| >0.5 and adjusted P-value (Padj) <0.01. For proteins, the threshold criteria were set at an adjusted P-value of <0.05. Visualization was performed using the EnhancedVolcano package (Blighe et al., 2018). Enrichment analysis of differentially expressed mRNAs (DEMs) and proteins (DEPros) was conducted using ClueGO (Bindea et al., 2009) in Cytoscape 3.10.1 (Shannon et al., 2003), with the reference database including the gene ontology (GO), kyoto encyclopedia of genes and genomes (KEGG) pathway, Reactome, and a statistical significance threshold set at *p* < 0.01. The enriched network was clustered based on groups and displayed with the shared genes between different pathways. Subsequently, the differentially expressed immune mRNAs (DEIMs) and proteins (DEIPros) were then identified by intersecting with the immune genes.

### 2.4 Selection of candidate DEIMs

The least absolute shrinkage and selective operator (LASSO) logistic regression model with 10-fold cross-validation was implemented using the glmnet R package (Friedman et al., 2010); the recursive feature elimination (RFE) algorithm with 10-fold cross-validation was performed using the Caret package (Kuhn, 2008). The receiver operating characteristic (ROC) curve, which reflects the prediction accuracy of each selected factor in sample classification, was calculated using the pROC (Robin et al., 2011) package in R. Factors with an area under the curve (AUC) greater than 0.78 were considered candidate DEIMs. The fitted model was subsequently constructed by combining a pair of non-integrated machine learning methods, the generalized linear model (GLM) and support vector machine (SVM), with a pair of integrated algorithms, the gradient boosting machine (GBM) and random forest (RF). The ROC curve and AUC were calculated using the Caret package.

### 2.5 External validation of key DEIMs in bulk and single-cell datasets

The expression profiles of the candidate DEIMs were validated using external gene expression datasets (GSE46224, GSE1145, GSE79962, GSE48166, GSE116250, and GSE120825). Candidate DEIMs expression was investigated in these datasets, followed by calculation and visualization of the differences between IHF and control samples using GraphPad Prism 8.0.2. Statistical significance was set at *p* < 0.05.

### 2.6 Construction of PPI network

The PPI network was predicted using the STRING database (Szklarczyk et al., 2015) and subsequently visualized using Cytoscape 3.10.1. Each node in the network was color-coded based on its differential expression level and the size of each node represented its degree within the network; the width of the edges indicated the betweenness centrality among the proteins. A subnetwork was identified using the MCODE algorithm (Bader and Hogue, 2003), from which a hub gene set was ranked and selected using the cytoHubba plugin (Chin et al., 2014). The ranking scores were then illustrated as a heatmap using complex heatmap package in R (Gu et al., 2016).

### 2.7 Western blotting and immunohistochemistry analysis

PTN expression was determined using Western blot analysis of samples collected from patients with ischemic cardiomyopathy and healthy hearts. The protein sample volume was calculated for each sample, and the protein to 4X loading buffer ratio was maintained at 1:3. Subsequently, the protein samples were denatured by boiling in a metal bath at 95 C for 5 min. After centrifugation of the denatured proteins and sample loading, the protein extracts were subjected to electrophoresis, followed by transfer onto polyvinylidene difluoride (PVDF) membranes for immunoblotting. For immunoblotting, primary antibodies targeting PTN and GAPDH at 1:2000 dilution, were used. Goat anti-rabbit IgG and Donkey anti-goat IgG were used as secondary antibodies. Collagen fibers were quantified using Masson’s trichrome staining. Immunohistochemistry (IHC) analysis was conducted for PTN in IHF samples. For IHC, the tissue sections were incubated with a primary rabbit polyclonal anti-PTN antibody at a dilution of 1:200, followed by detection using a goat anti-rabbit IgG secondary antibody.

### 2.8 Statistical analysis

Statistical analyses were performed using the R 4.2.3 and GraphPad Prism 8.0.2 software packages. Data are presented as mean ± standard deviation (SD) when a normal distribution was followed. Pearson’s correlation analysis was used to examine the relationships between variables, whereas Student’s t-test was used to compare the differences between the two groups. Spearman correlation analysis and the Mann-Whitney *U* test were used for non-normally distributed data to assess correlations and group differences, respectively. Statistical significance was denoted by *p < 0.05, **p < 0.01, ***p < 0.001, and ****p < 0.0001.

## 3 Results

### 3.1 Differential expression and enrichment analysis highlight immune-related characteristics in IHF

**FIGURE 2:**
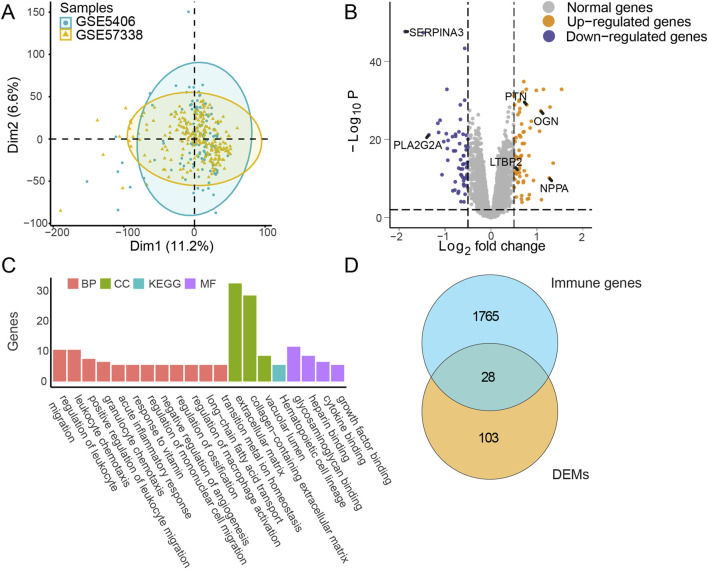
Differential expression and enrichment analysis in IHF. **(A)** Principal component analysis (PCA): Data from merged datasets GSE5406_GSE57338, with batch effects removed, projected onto two dimensions (Dim1 and Dim2). Blue markers represent sample groups from GSE5406, and yellow markers represent sample groups from GSE57338. **(B)** Differential expression analysis: Display of log2 fold change (|log2FC|) and -Log10 P-values (Padj) for each mRNA comparing IHF to non-failure samples. The threshold of |log2FC| >0.5 and Padj <0.01 were used to obtain DEMs. Gray markers indicate normally expressed mRNAs, blue markers indicate downregulated mRNAs, and yellow markers indicate upregulated mRNAs. Several key gene names were marked, including SERPINA3, PLA2G2A, PTN, OGN, LTBP2 and NPPA. **(C)** Pathway enrichment analysis: Bar plot representing enriched pathways among DEMs. Red, green, and purple markers highlight annotations from biological process (BP), cellular component (CC), and molecular function (MF) in GO, respectively. Blue markers indicate annotations from KEGG analysis. **(D)** Intersection analysis: Visualization of the overlap between DEMs and immune-related genes. IHF, ischemic heart failure; DEMs, Differentially expressed mRNAs; GO, Gene ontology; KEGG, Kyoto encyclopedia of genes and genomes.

The GSE5406 and GSE57338 datasets were merged, and batch effects were removed to generate a highly normalized expression profile for subsequent analysis (Figure 2A). Based on the clustering results, marked molecular differences emerged between the IHF and non-IHF samples. Subsequently, 131 differentially expressed mRNAs (DEMs) were identified using a threshold of |log2FC| >0.5 and Padj <0.01 (Figure 2B). The top 10 DEMs are listed in Supplementary Table S2.

Enrichment analysis of the DEMs indicated that multiple pathways, including the acute inflammatory response and regulation of leukocyte migration, achieved high enrichment scores, emphasizing the pivotal role of immune responses during IHF progression (Figure 2C). The upregulation of fibrosis-related pathways was observed, which is suggesting that cardiac remodeling plays a critical role in IHF. To investigate these important immune characteristics, we intersected the identified DEMs with an immune gene list sourced from ImmPort (https://www.immport.org/). This analysis identified 28 differentially expressed immune mRNAs (DEIMs), which were evaluated for their potential as biomarkers in subsequent steps (Figure 2D).

### 3.2 Feature selection and validation of candidate DEIMs using machine learning techniques

The merged dataset, characterized by its substantial size and normality, demonstrated consistent characteristics, thereby offering promising prospects for machine learning applications. RFE and LASSO regression were used as representative methods of feature selection to screen candidate DEIMs.

**FIGURE 3:**
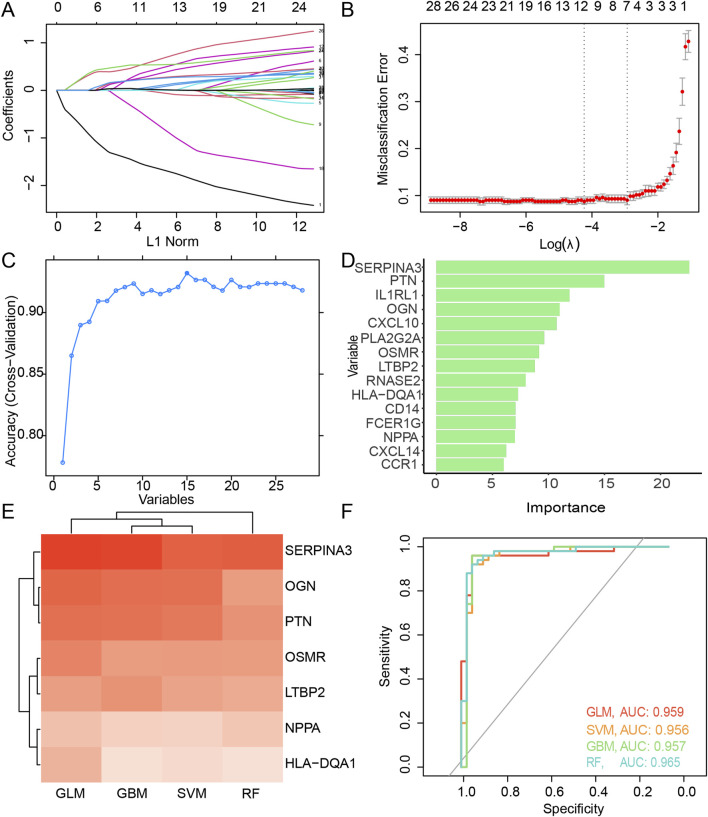
Feature selection of the candidate DEIMs. **(A)** Variation coefficient for each variable during the LASSO regression. **(B)** Cross-validation for penalty parameter selection. **(C)** Accuracy variation with the count of total variables during RFE. **(D)** Importance of each optimal variable screened using RFE. **(E)** Prediction performance and AUC value of each intersected candidate DEIM using four algorithms, including GLM, SVM, GBM, and RF. **(F)** ROC of the candidate DEIM set, with each color representing GLM, SVM, GBM, and RF, respectively. DEIMs, Differentially expressed immune mRNAs; LASSO, Least absolute shrinkage and selective operator; RFE, Recursive feature elimination; GLM, Generalized linear model; SVM, Supporting vector machine; GBM, Gradient boost machine; RF, Random forest; ROC, Receiver operating characteristic; AUC, Area under the curve.

By performing weight calculations for each factor and constructing a fit model with the minimum lambda value (Figures 3A, B), 10 DEIMs (HLA-DQA1, SERPINA3, CXCL14, PLA2G2A, LTBP2, SPP1, PTN, OSMR, OGN, NPPA) were identified using LASSO regression. When using RFE as the selection method, 17 important variables, SERPINA3, PTN, IL1RL1, OGN, CXCL10, PLA2G2A, OSMR, LTBP2, RNASE2, HLA-DQA1, NPPA, FCER1G, CD14, CCR1, CXCL14, SPP1, and SLC11A1, were identified when both the accuracy and kappa score reached their first peak (Figure 3C). The importance of each variable was calculated and ranked accordingly (Figure 3D). Proteins such as SERPINA3, PTN, and OGN have been reported to be highly correlated with cardiac fibrosis (Pál et al., 2023).

Seven candidate hub DEIMs (NPPA, LTBP2, OSMR, OGN, HLA-DQA1, PTN, and SERPINA3) were identified as important because they were commonly screened using the LASSO regression and RFE algorithms (Supplementary Table S3). Subsequently, prediction models for disease diagnosis were constructed using the four algorithms (GLM, SVM, GBM, and RF) for each mRNA (Figure 3E). When considering single-molecule predictions, the GLM demonstrated the highest accuracy among all the algorithms, with SERPINA3, OGN, and PTN showing the most precise results. The overall performance of these candidate DEIMs was visualized using ROC curves generated by the GLM, SVM, GBM, and RF algorithms (Figure 3F). The total accuracy remained consistent across the different algorithms; all AUCs exceeded 0.9, suggesting high precision in predicting disease outcomes using these candidate DEIMs.

### 3.3 External validation of candidate DEIMs using multiple bulk RNA-seq datasets

**FIGURE 4:**
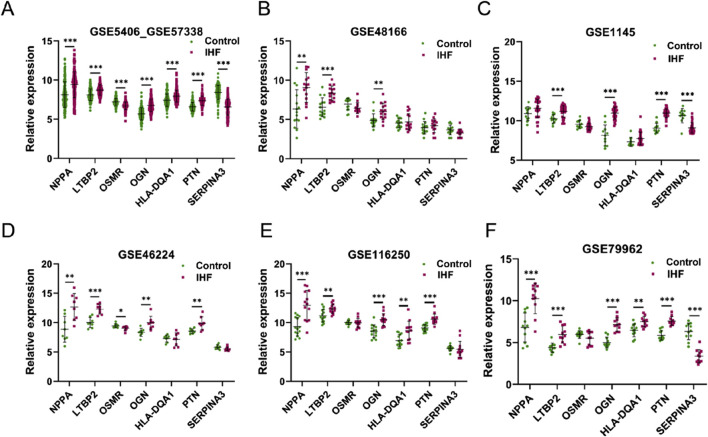
External validation of candidate DEIMs. **(A–F)** The expression levels of the seven candidate mRNAs (NPPA, LTBP2, OSMR, OGN, HLA-DQA1, PTN and SERPINA3) were assessed in six datasets (GSE5406_GSE57338, GSE48166, GSE1145, GSE46224, GSE116250, and GSE79962). The term “GSE5406_GSE57338”refers to the combined data from GSE5406 and GSE57338. IHF refers to patients with ischemic heart failure, whereas control refers to samples from healthy individuals.

The expression of the seven genes identified using the four algorithms was investigated using multiple external datasets (GSE48166, GSE1145, GSE46224, GSE116250, and GSE79962) for validation (Figures 4B–F). Among the candidate DEIMs, OGN was consistently upregulated in all external datasets, whereas LTBP2 was upregulated in five datasets, and NPPA was upregulated in four datasets. PTN was dysregulated in three additional datasets, whereas OSMR, HLA-DQA1, and SERPINA3 were less regulated in IHF. Ultimately, four DEIMs (OGN, NPPA, LTBP2, and PTN) were identified as hub genes with dysregulated expression in IHF (Figure 4).

### 3.4 ScRNA-seq data indicate cell-specific expression patterns of key DEIMs

**FIGURE 5:**
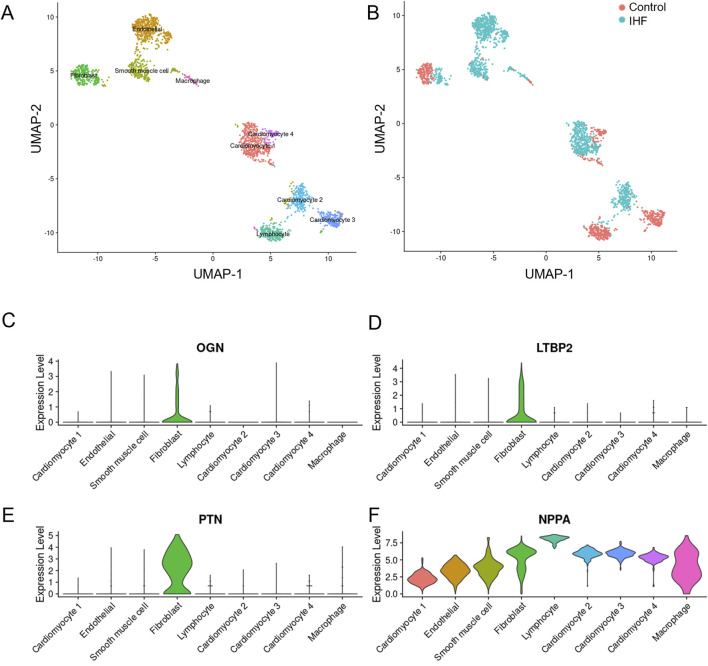
The single-cell dataset analysis of four key DEIMs was conducted in IHF and non-failure controls. **(A)** The UMAP plot indicates the separation of 4,933 cells into nine distinct subtypes. **(B)** Each cell population was accompanied by a corresponding sample statement. **(C–F)** Violin plots effectively demonstrated the cell-specific expression patterns of the four key DEIMs (OGN, LTBP2, PTN and NPPA). DEIMs, Differentially expressed immune mRNAs.

Single-cell analysis was conducted to elucidate the function of each hub DEIM and its underlying mechanisms. Feature dimension reduction facilitated the classification of 4,993 cells into nine distinct clusters (Figure 5A). Marked differences were observed between the cell populations of the non-failed and IHF samples (Figure 5B), with each sample type corresponding to a specific cell cluster.

In contrast to the control group, IHF samples exhibited substantial differentiation into endothelial cells, smooth muscle cells, lymphocytes, and cardiomyocyte types I and II. Fibroblasts and cardiomyocytes were further classified into IHF-related and IHF-non-related subtypes, which are believed to play crucial roles in myocardial fibrosis and myocardial fibroblast transformation. Compared to NPPA, the three hub DEIMs demonstrated cell-specific expression. PTN was highly expressed in fibroblasts, indicating its potential role in regulating fibrosis (Figures 5C–F). Consequently, OGN, LTBP2, and PTN were identified as hub DEIMs.

### 3.5 Integrated analysis of hub DEIMs at the transcriptomic and proteomic levels

**FIGURE 6:**
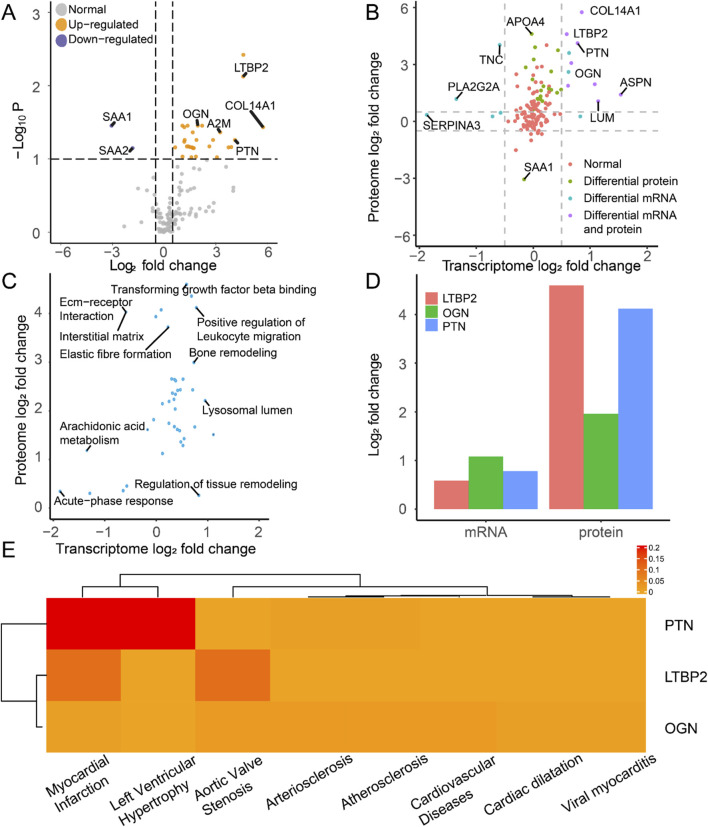
Molecular expression and functional enrichment of hub DEIMs at the transcriptomic and proteomic levels. **(A)** The volcano plot of protein distribution by comparing IHF samples with non-failure samples, based on the log2 fold change and the negative log10 P-values of each protein. The grey icon represents the normal protein, the blue icon represents the downregulated protein, and the yellow icon indicates the upregulated protein. **(B)** The common differentially expressed molecules at the transcriptomic and proteomic levels. **(C)** Common enriched pathway at the transcriptomic and proteomic levels. **(D)** The log2 fold change of the hub molecules at the mRNA and protein levels. **(E)** The disease-specific score of the hub immune molecule is indicated by color, which represents the DisGeNET ranking for each molecule.

After extracting the proteomic data, we identified 29 upregulated and 2 downregulated proteins as DEPros (Figure 6A, Supplementary Table S4). Enrichment analysis indicated the upregulation of the humoral immune response, extracellular organization, and fibrosis collagen pathway (Supplementary Figure S1A), prompting us to focus our research on the immune landscape at the proteomic level. The differentially expressed molecules (Figure 6B) and enriched pathways (Figure 6C) at the transcriptomic and proteomic levels were integrated in the overall analysis. By conducting transcriptomic and proteomic analyses, three hub proteins, PTN, LTBP2, and OGN, were identified based on their significant differential expression characteristics at both the transcriptomic and proteomic levels (Figure 6D).

We examined the disease-specific expression patterns of these three proteins (Figure 6E). Compared with LTBP2 and OGN, PTN exhibited enrichment in myocardial infarction, which is the primary cause of cardiac ischemia and subsequent heart failure. These findings suggest the specific expression of PTN in ischemia-related heart diseases and highlight its clinical significance over other biomarkers.

### 3.6 Screening the hub immune protein on the basis of network analysis

After determining the differential expression of transcription levels, we proceeded to analyze protein expression levels. The study focused on investigating the immune landscape at the proteomic level (Supplementary Figure S1A). Despite the effectiveness of machine learning in handling large datasets, it falls short of fully capturing the information encompassed within the proteome profile. Consequently, we developed a novel analysis pipeline for -omics data in limited quantities by implementing multilevel cleansing and amplification techniques. A primary PPI network was constructed with all proteins in the dataset using STRING. However, due to the inclusion of references from diverse species and tissues, there was a possibility of redundant and nonspecific information within the database. Therefore, we conducted multiple data-cleansing procedures based on network analysis. The core module of immune-related proteins was extracted by considering the differentially-expressed, immune, and highly influential proteins involved and cluster scores. Based on the results obtained using CytoHubba and MCODE (Supplementary Figure S1B), a differentially expressed immune module was identified as the primary contributor to the entire network, wherein most proteins were significantly associated with fibrosis (Supplementary Figure S1C). Using Humanbase prediction, interactions within the subnetwork were extensively and specifically expanded, whereas the expression patterns of each protein were validated based on comprehensive integrated data (Supplementary Figure S2A). Ultimately, differentially expressed immune proteins (DEIPros) displaying highly co-expressed characteristics were identified, of which LTBP2 and PTN were detected in the proteomic profile and were considered to be pivotal DEIPros. Furthermore, the hub DEIPros were utilized to predict the pathway-specific network using HumanBase. A strong correlation was observed between fibroblast proliferation and ECM organization (Supplementary Figures S2B, C). As an DEIPro, PTN is involved in multiple interactions with fibrosis or collagen factors, suggesting its potential role as a target for cardiac remodeling.

### 3.7 Upregulated expression of PTN in the left ventricle of IHF samples

**FIGURE 7:**
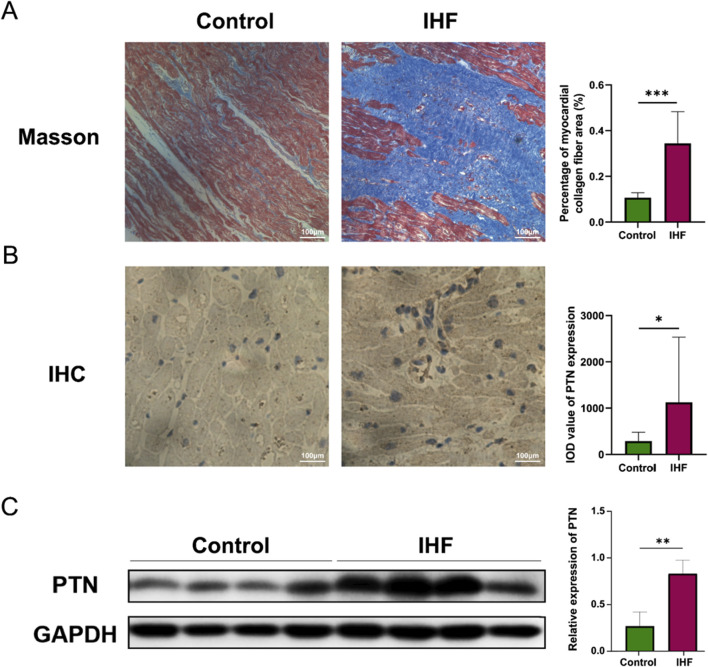
The expression of PTN was upregulated in IHF. **(A)** The Masson’s staining of myocardial collagen fibers was performed in IHF and control samples. The corresponding graphs present the analysis of the percentage of myocardial collagen fiber area. **(B)** IHC staining and **(C)** Western blotting indicate an increased expression of PTN in IHF samples compared to controls. Densitometric analysis and quantification of integrated optical density (IOD) were presented in the corresponding graphs. Scale bar: 100 μm. The Western blotting presented in this figure has undergone brightness adjustments to the background for improved clarity, without altering the relative intensities of the bands. P- values were calculated using Mann–Whitney *U* test and Student’s t-test. *p < 0.05, 0.05 < **p < 0.001, ***p < 0.001. IHF, Ischemic heart failure; IHC, Immunohistochemistry.

To further validate these findings, we conducted a wet experiment to investigate PTN protein expression in the samples. Patients with IHF exhibited substantially more severe left ventricular tissue fibrosis than the healthy controls (Figure 7A). Semi-quantitative IHC analysis revealed a substantial increase in PTN levels in IHF samples, particularly in the intercellular stroma (Figure 7B). Western blotting demonstrated a substantial elevation of PTN expression in IHF samples compared to healthy controls (Figure 7C). These results indicate a concordant upregulation of PTN protein expression in IHF samples.

## 4 Discussion

IHF is a complex and heterogeneous disease that presents significant challenges in diagnosis and treatment. In this study, we conducted a multi-omics analysis to identify novel potential biomarkers for IHF. By employing differential expression analysis, enrichment analysis, machine learning, and network analysis across transcriptomic and proteomic levels, several hub molecules were identified. Among them, PTN has shown significant upregulation in multiple datasets and the activation of various molecules associated with dysplastic cardiac remodeling. By synthesizing these data with clinical findings, we have identified PTN as a potential therapeutic and diagnostic target, which was verified by IHC and Western blotting. These findings provide valuable insights into the molecular mechanisms underlying IHF. The current study proposes a pipeline for omics analysis that is free from data quantity constraints, representing a refreshing departure from traditional methodologies, particularly in proteomic studies.

Clinical studies have highlighted the importance of preventing dysplastic remodeling in idiopathic pulmonary fibrosis. Although several immune targets, such as MMP/TIMP, TGFB, and IFN-γ, have been developed for IHF treatment, they lack broad-spectrum efficacy as reliable indicators (Romeo et al., 2023; Zhang et al., 2022). In the present study, we identified three mRNAs and proteins (OGN, LTBP2, and PTN) as potential biomarkers and key players in fibrosis. The roles of OGN and LTBP2 in IHF have been previously investigated (Romeo et al., 2023). However, the involvement of PTN in fibrosis progression has rarely been reported in previous research or comprehensively illustrated within related pathways (Zhang et al., 2022). Therefore, we conducted a systematic investigation to examine the participation and clinical value of PTN.

Based on the findings of this case, PTN may function as a fibrosis-related factor by mediating the autocrine regulation of fibroblasts and inducing collagen secretion. Initially identified as a neuron growth factor, subsequent studies revealed multiple functions of PTN, including the promotion of vascular endothelial growth and fibroblast proliferation (Gu et al., 2007; Perez-Pinera et al., 2007). Molecular evidence also suggests its involvement in diverse signaling pathways, activating downstream factors, such as ALK and PI3K/AKT receptors, for various biological processes (Perez-Pinera et al., 2008). In fibrosis caused by mitral regurgitation, PTN is upregulated in the myocardial transcriptome, suggesting its role in cardiac remodeling (Duggal et al., 2023). Based on these findings, we propose that PTN could be synthesized during heart failure to regulate collagen secretion, leading to overall extracellular matrix deposition. Facilitation of fibroblast growth by PTN may contribute to their transformation into cardiac fibroblasts, which is another crucial factor driving the pathological remodeling of cardiac muscle.

This systematic strategy has been widely applied in recent studies, including the integration of multiple -omics datasets and concordance with various aspects of biological activities. Owing to the complex interactions between metabolic remodeling and immune regulation in heart failure, relying solely on immune indicators may not fully capture the comprehensive profile of disease development. Although machine-learning methods have shown promising results in data screening and prediction, their performance needs to be validated using a larger sample size. Accurately classifying different disease types is challenging because of the heterogeneity observed in the mechanisms of idiopathic heart failure. To address these issues, future research should adopt a more systematic approach by considering distinct modules, including metabolism and immune responses, while integrating -omics data to construct a multidimensional model.

## Data Availability

The datasets presented in this study can be found in online repositories. The names of the repository/repositories and accession number(s) can be found in the article/Supplementary Material.

## References

[B1] Bader G. D. Hogue C. W. (2003). An automated method for finding molecular complexes in large protein interaction networks. BMC Bioinforma. 4, 2. 10.1186/1471-2105-4-2 PMC149346 12525261

[B2] Barallobre-Barreiro J. Radovits T. Fava M. Mayr U. Lin W. Y. Ermolaeva E. (2021). Extracellular matrix in heart failure: role of ADAMTS5 in proteoglycan remodeling. Circulation 144, 2021–2034. 10.1161/CIRCULATIONAHA.121.055732 34806902 PMC8687617

[B3] Barrett T. Wilhite S. E. Ledoux P. Evangelista C. Kim I. F. Tomashevsky M. (2013). NCBI GEO: archive for functional genomics data sets--update. Nucleic acids Res. 41, D991–D995. 10.1093/nar/gks1193 23193258 PMC3531084

[B4] Basak T. Varshney S. Akhtar S. Sengupta S. (2015). Understanding different facets of cardiovascular diseases based on model systems to human studies: a proteomic and metabolomic perspective. J. proteomics 127, 50–60. 10.1016/j.jprot.2015.04.027 25956427

[B5] Bindea G. Mlecnik B. Hackl H. Charoentong P. Tosolini M. Kirilovsky A. (2009). ClueGO: a cytoscape plug-in to decipher functionally grouped gene ontology and pathway annotation networks. Bioinforma. Oxf. Engl. 25, 1091–1093. 10.1093/bioinformatics/btp101 PMC2666812 19237447

[B6] Blighe K. Rana S. Lewis M. (2018). EnhancedVolcano: publication-ready volcano plots with enhanced colouring and labeling. Bioconductor. 10.18129/B9.bioc.EnhancedVolcano

[B7] Bozkurt B. Coats A. J. S. Tsutsui H. Abdelhamid C. M. Adamopoulos S. Albert N. (2021). Universal definition and classification of heart failure: a report of the heart failure society of America, heart failure association of the European society of cardiology, Japanese heart failure society and writing committee of the universal definition of heart failure: endorsed by the Canadian heart failure society, heart failure association of India, cardiac society of Australia and New Zealand, and Chinese heart failure association. Eur. J. Heart Fail. 23, 352–380. 10.1002/ejhf.2115 33605000

[B8] Chaffin M. Papangeli I. Simonson B. Akkad A. D. Hill M. C. Arduini A. (2022). Single-nucleus profiling of human dilated and hypertrophic cardiomyopathy. Nature 608, 174–180. 10.1038/s41586-022-04817-8 35732739 PMC12591363

[B9] Chin C. H. Chen S. H. Wu H. H. Ho C. W. Ko M. T. Lin C. Y. (2014). CytoHubba: identifying hub objects and sub-networks from complex interactome. BMC Syst. Biol. 8, S11. 10.1186/1752-0509-8-S4-S11 25521941 PMC4290687

[B10] Cunningham F. Allen J. E. Allen J. Alvarez-Jarreta J. Amode M. R. Armean I. M. (2022). Ensembl 2022. Nucleic acids Res. 50, D988–D995. 10.1093/nar/gkab1049 34791404 PMC8728283

[B11] Duggal N. M. Lei I. Wu X. Aaronson K. D. Pagani F. D. Lam H. Y. (2023). Mitral regurgitation severity at left ventricular assist device implantation is associated with distinct myocardial transcriptomic signatures. J. Thorac. Cardiovasc. Surg. 161, 141–152.e1. 10.1016/j.jtcvs.2021.08.061 PMC11217920 34689984

[B12] Feng Q. Li Q. Zhou H. Sun L. Lin C. Jin Y. (2023). The role of major immune cells in myocardial infarction. Front. Immunol. 13, 1084460. 10.3389/fimmu.2022.1084460 36741418 PMC9892933

[B13] Friedman J. Hastie T. Tibshirani R. (2010). Regularization paths for generalized linear models via coordinate descent. J. Stat. Softw. 33, 1–22. 10.18637/jss.v033.i01 20808728 PMC2929880

[B14] Gharbin J. Winful A. Hassan M. A. Bajaj S. Batta Y. Alebna P. (2023). Differences in the clinical outcome of ischemic and nonischemic cardiomyopathy in heart failure with concomitant opioid use disorder. Curr. problems Cardiol. 48, 101609. 10.1016/j.cpcardiol.2023.101609 36690309

[B15] Gu D. Yu B. Zhao C. Ye W. Lv Q. Hua Z. (2007). The effect of pleiotrophin signaling on adipogenesis. FEBS Lett. 581, 382–388. 10.1016/j.febslet.2006.12.043 17239862

[B16] Gu Z. Eils R. Schlesner M. (2016). Complex heatmaps reveal patterns and correlations in multidimensional genomic data. Bioinforma. Oxf. Engl. 32, 2847–2849. 10.1093/bioinformatics/btw313 27207943

[B17] Hao Y. Hao S. Andersen-Nissen E. Mauck W. M. 3rd Zheng S. Butler A. (2021). Integrated analysis of multimodal single-cell data. Cell 184, 3573–3587.e29. 10.1016/j.cell.2021.04.048 34062119 PMC8238499

[B18] Kanapeckaitė A. Burokienė N. (2021). Insights into therapeutic targets and biomarkers using integrated multi-'omics' approaches for dilated and ischemic cardiomyopathies. Integr. Biol. quantitative Biosci. nano macro 13, 121–137. 10.1093/intbio/zyab007 33969404

[B19] Kim D. Landmead B. Salzberg S. L. (2015). HISAT: a fast spliced aligner with low memory requirements. Nat. Methods 12, 357–U121. 10.1038/Nmeth.3317 25751142 PMC4655817

[B20] Kuhn M. J. J. (2008). Building predictive models in R using the caret package. J. Statistcal Softw. 28, 1–26. 10.18637/jss.v028.i05

[B21] Li M. Parker B. L. Pearson E. Hunter B. Cao J. Koay Y. C. (2020). Core functional nodes and sex-specific pathways in human ischaemic and dilated cardiomyopathy. Nat. Commun. 11, 2843. 10.1038/s41467-020-16584-z 32487995 PMC7266817

[B22] Love M. I. Huber W. Anders S. (2014). Moderated estimation of fold change and dispersion for RNA-seq data with DESeq2. Genome Biol. 15, 550. 10.1186/s13059-014-0550-8 25516281 PMC4302049

[B23] Nian W. Huang Z. Fu C. (2023). Immune cells drive new immunomodulatory therapies for myocardial infarction: from basic to clinical translation. Front. Immunol. 14, 1097295. 10.3389/fimmu.2023.1097295 36761726 PMC9903069

[B24] Pál K. Mănescu I. B. Lupu S. Dobreanu M. (2023). Emerging biomarkers for predicting clinical outcomes in patients with heart disease. Life Basel, Switz. 13, 230. 10.3390/life13010230 PMC9864006 36676179

[B25] Perez-Pinera P. Berenson J. R. Deuel T. F. (2008). Pleiotrophin, a multifunctional angiogenic factor: mechanisms and pathways in normal and pathological angiogenesis. Curr. Opin. Hematol. 15, 210–214. 10.1097/MOH.0b013e3282fdc69e 18391787

[B26] Perez-Pinera P. Chang Y. Deuel T. F. (2007). Pleiotrophin, a multifunctional tumor promoter through induction of tumor angiogenesis, remodeling of the tumor microenvironment, and activation of stromal fibroblasts. Cell cycleGeorget. Tex 6, 2877–2883. 10.4161/cc.6.23.5090 18156802

[B27] Polyakova V. Loeffler I. Hein S. Miyagawa S. Piotrowska I. Dammer S. (2011). Fibrosis in endstage human heart failure: severe changes in collagen metabolism and MMP/TIMP profiles. Int. J. Cardiol. 151, 18–33. 10.1016/j.ijcard.2010.04.053 20546954

[B28] Rao M. Wang X. Guo G. Wang L. Chen S. Yin P. (2021). Resolving the intertwining of inflammation and fibrosis in human heart failure at single-cell level. Basic Res. Cardiol. 116, 55. 10.1007/s00395-021-00897-1 34601654

[B29] Ritchie M. E. Phipson B. Wu D. Hu Y. Law C. W. Shi W. (2015). Limma powers differential expression analyses for RNA-sequencing and microarray studies. Nucleic acids Res. 43, e47. 10.1093/nar/gkv007 25605792 PMC4402510

[B30] Robin X. Turck N. Hainard A. Tiberti N. Lisacek F. Sanchez J. C. (2011). pROC: an open-source package for R and S+ to analyze and compare ROC curves. BMC Bioinforma. 12, 77. 10.1186/1471-2105-12-77 PMC3068975 21414208

[B31] Romeo F. J. Mavropoulos S. A. Ishikawa K. (2023). Progress in clinical gene therapy for cardiac disorders. Mol. diagnosis and Ther. 27, 179–191. 10.1007/s40291-022-00632-z PMC10023344 36641770

[B32] Rong Z. Chen H. Zhang Z. Zhang Y. Ge L. Lv Z. (2022). Identification of cardiomyopathy-related core genes through human metabolic networks and expression data. BMC genomics 23, 47. 10.1186/s12864-021-08271-0 35016605 PMC8753885

[B33] Savarese G. Becher P. M. Lund L. H. Seferovic P. Rosano G. M. C. Coats A. J. S. (2023). Global burden of heart failure: a comprehensive and updated review of epidemiology. Cardiovasc. Res. 118, 3272–3287. 10.1093/cvr/cvac013 35150240

[B34] Shannon P. Markiel A. Ozier O. Baliga N. S. Wang J. T. Ramage D. (2003). Cytoscape: a software environment for integrated models of biomolecular interaction networks. Genome Res. 13, 2498–2504. 10.1101/gr.1239303 14597658 PMC403769

[B35] Szklarczyk D. Franceschini A. Wyder S. Forslund K. Heller D. Huerta-Cepas J. (2015). STRING v10: protein-protein interaction networks, integrated over the tree of life. Nucleic acids Res. 43, D447–D452. 10.1093/nar/gku1003 25352553 PMC4383874

[B36] Zhang Y. Zhang J. J. Feng D. Y. Zhou H. Gui Z. P. Zheng M. (2022). IRF1/ZNF350/GPX4-mediated ferroptosis of renal tubular epithelial cells promote chronic renal allograft interstitial fibrosis. Free Radic. Bio Med. 193, 579–594. 10.1016/j.freeradbiomed.2022.11.002 36356714

[B37] Zhou Y. Zhou B. Pache L. Chang M. Khodabakhshi A. H. Tanaseichuk O. (2019). Metascape provides a biologist-oriented resource for the analysis of systems-level datasets. Nat. Commun. 10, 1523. 10.1038/s41467-019-09234-6 30944313 PMC6447622

