# Supplementary material for: Integrated multi-omics analysis describes immune profiles in ischemic heart failure and identifies PTN as a novel biomarker
Source: Front Mol Biosci. 2024 Dec 11;11:1524827. doi: 10.3389/fmolb.2024.1524827 (PMC11668632; doi:10.3389/fmolb.2024.1524827)
Supplement: Supplementary file 1 [file DataSheet1.docx]

Supplementary Material

# Supplementary Figures and Tables

## Supplementary Figures


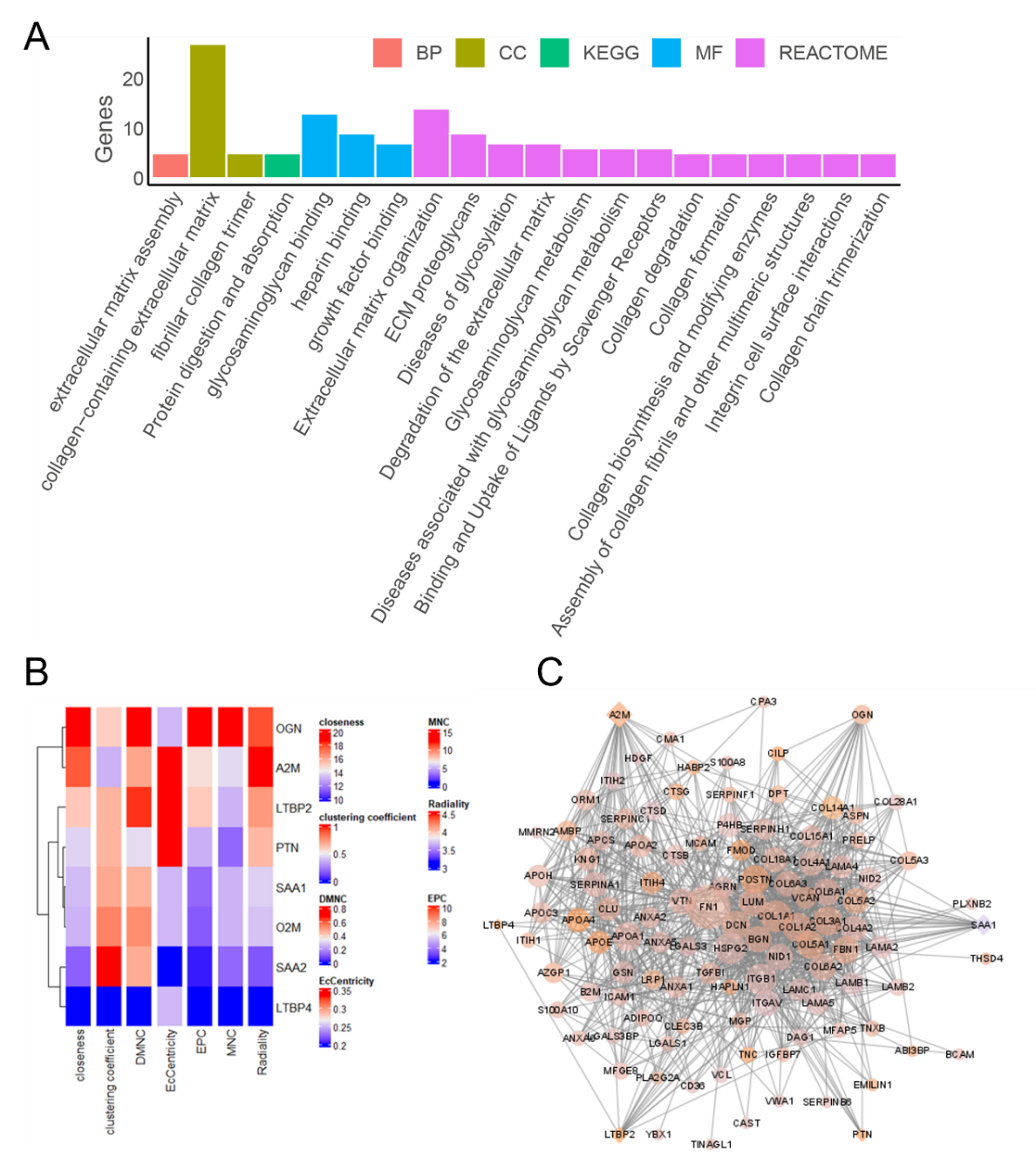


**Supplementary Figure S1. Significant immune characteristics and network analysis based on proteomic data.**

(A) Pathway enrichment analysis. The bar plot illustrates the enriched pathways among DEPros. Red, yellow, and blue markers highlight annotations from biological process (BP), cellular component (CC), and molecular function (MF) in GO, respectively. Green markers indicate annotations from KEGG analysis. Pink markers indicate annotations from the REACTOME database. (B) CytoHubba analysis. The heatmap displays scores for each candidate hub DEIPros using various algorithms, including closeness, clustering coefficient, DMNC, EcCentricity, EPC, MNC, and radiality. (C) Protein-protein interacting network of a sub-module selected using MCODE. DEPros, Differentially expressed proteins; DEIPros, Differentially expressed immune proteins; GO, Gene ontology; KEGG, Kyoto encyclopedia of genes and genomes.


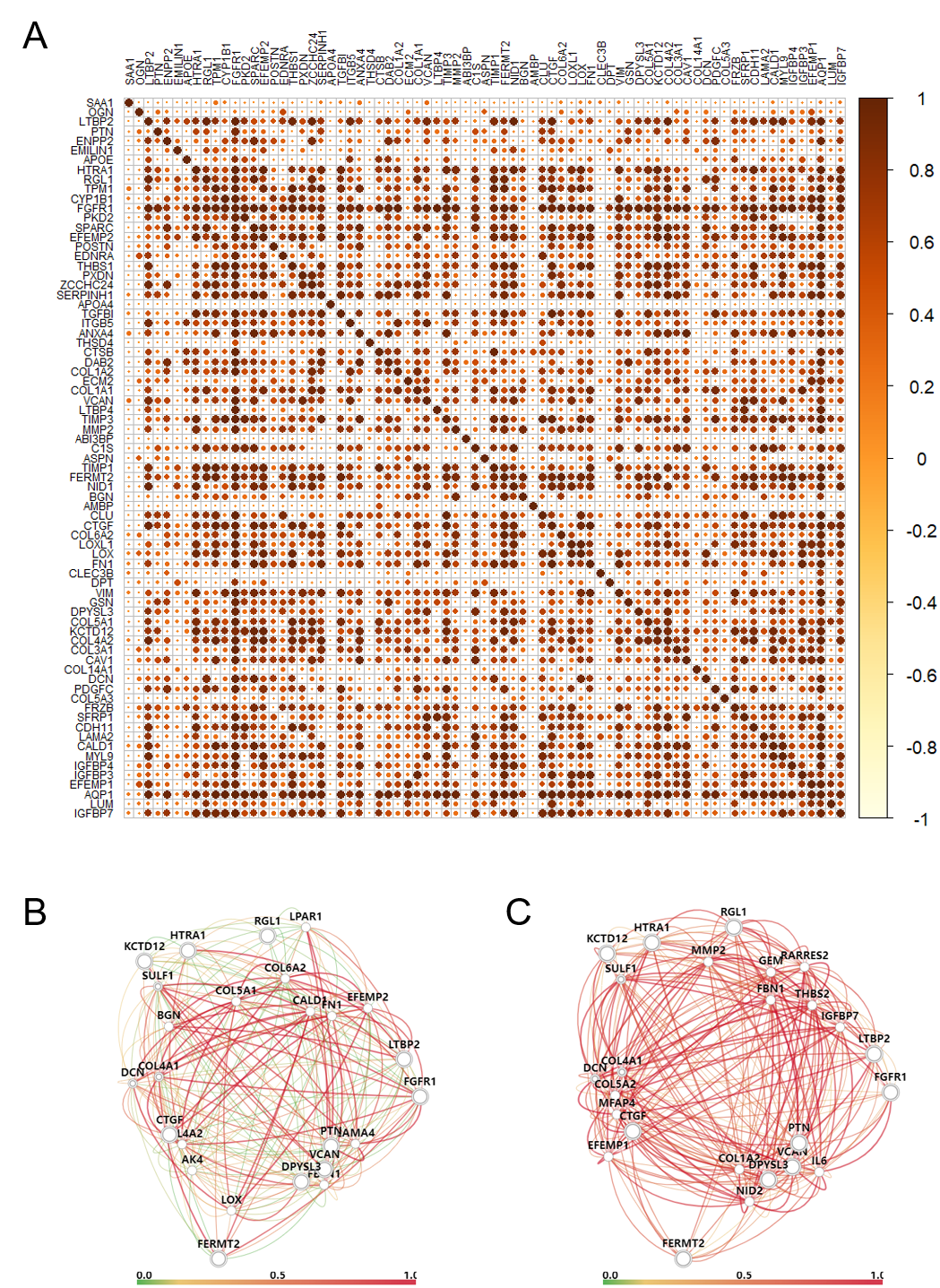


**Supplementary Figure S2. Cardio-specific expression profile of candidate immune protein.**

(A) Correlation analysis. A heatmap is used to represent the correlation between each predicted immune protein, with color indicating the strength of the correlation. (B) Correlation network of hub immune proteins involved in fibroblast growth process. Each node represents a protein and each edge represents the correlation between two nodes. (C) Correlation network of hub immune proteins involved in the JAK/STAT signal pathway. Each node represents a protein, and each edge represents the correlation between two nodes.

## Supplementary Tables

| Dataset | Disease | Data type | Sample |
| --- | --- | --- | --- |
| GSE5406 | IHF | Array | 16 controls, 108 IHFs from LV |
| GSE57338 | IHF | Array | 136 controls, 95 IHFs from LV |
| GSE48166 | IHF | RNA-seq | 15 controls, 15 IHFs from LV |
| GSE1145 | IHF | RNA-seq | 11 controls, 31 IHFs from LV |
| GSE46224 | IHF | RNA-seq | 8 controls, 8 IHFs from LV |
| GSE116250 | IHF | RNA-seq | 14 controls, 13 IHFs from LV |
| GSE79962 | IHF | RNA-seq | 11 controls, 11 IHFs from LV |
| GSE121893 | IHF | scRNA-seq | 2 controls, 2 IHFs from LV and LA |

## Supplementary Table S1. Details of all the dataset obtained from GEO database.

## Notes:LV: left ventricle; LA: left atrial.

**Supplementary Table S2. List of top 10 DEMs detected in this study.**

| mRNA | Log2FC | P-Value | Adj.p.value |
| --- | --- | --- | --- |
| NPPA | 1.27 | 0.00 | 0.00 |
| OGN | 1.08 | 0.00 | 0.00 |
| PTN | 0.78 | 0.00 | 0.00 |
| LTBP2 | 0.59 | 0.00 | 0.00 |
| CXCL14 | 0.59 | 0.00 | 0.00 |
| NPR3 | 0.57 | 0.00 | 0.00 |
| CXCL10 | 0.55 | 0.00 | 0.00 |
| HSPA2 | 0.52 | 0.00 | 0.00 |
| HLA-DQA1 | 0.51 | 0.00 | 0.00 |
| CCR1 | -0.50 | 0.00 | 0.00 |

**Supplementary Table S3. Intersection of candidate DEIMs screened by LASSO regression and the RFE algorithm.**

| Algorithm | Candidate DEIMs |
| --- | --- |
| LASSO | HLA-DQA1, SERPINA3, CXCL14, PLA2G2A, LTBP2, SPP1, PTN, OSMR, OGN, NPPA |
| RFE | SERPINA3, PTN, IL1RL1, OGN, CXCL10, PLA2G2A, OSMR, LTBP2, RNASE2, HLA-DQA1, NPPA, FCER1G, CD14, CCR1, CXCL14, SPP1, SLC11A1 |
| Intersection | NPPA, LTBP2, OSMR, OGN, HLA-DQA1, PTN, SERPINA3 |

**Supplementary Table S4. List of top 10 DEPros detected in this study.**

| Protein | Log2FC | P-Value | Adj.p.value |
| --- | --- | --- | --- |
| COL14A1 | 5.75 | 0.00 | 0.04 |
| APOA4 | 4.61 | 0.00 | 0.00 |
| LTBP2 | 4.60 | 0.00 | 0.01 |
| PTN | 4.12 | 0.00 | 0.06 |
| APOE | 3.91 | 0.01 | 0.07 |
| LTBP4 | 3.75 | 0.01 | 0.07 |
| A2M | 3.26 | 0.00 | 0.04 |
| POSTN | 3.07 | 0.02 | 0.10 |
| EMILIN1 | 2.85 | 0.00 | 0.06 |
| ABI3BP | 2.63 | 0.01 | 0.07 |

**Supplementary Table S5. List of abbreviations.**

| Abbreviations | Full names |
| --- | --- |
| HF | Heart failure |
| IHF | Ischemic heart failure |
| PTN | Pleiotrophin |
| MFBs | Myofibroblasts |
| ECM | Extracellular matrix |
| snRNA-seq | Single-nucleus sequencing |
| scRNA-seq | Single-cell RNA sequencing |
| PPI | Protein-protein interaction |
| GEO | Gene expression omnibus database |
| NCBI | National center for biotechnology information |
| ENA | European nucleotide archive |
| DEMs | Differentially expressed mRNAs |
| DEPros | Differentially expressed proteins |
| DEIMs | Differentially expressed immune mRNAs |
| DEIPros | Differentially expressed immune proteins |
| LASSO | Least absolute shrinkage and selective operator |
| RFE | Recursive feature elimination |
| GLM | Generalized linear model |
| SVM | Supporting vector machine |
| GBM | Gradient boost machine |
| RF | Random forest |
| NPPA | Natriuretic peptide type A |
| LTBP2 | Latent transforming growth factor beta binding protein 2 |
| OGN | Osteoglycin |
| GO | Gene ontology |
| KEGG | Kyoto encyclopedia of genes and genomes |
| ROC | Receiver operating characteristic |
| AUC | Area under the curve |
| PVDF | Polyvinylidene difluoride |
| IHC | Immunohistochemistry |
| BP | Biological process |
| CC | Cellular component |
| MF | Molecular function |
| IOD | Integrated optical density |
